
JOURNAL INFORMATION
==============================
NLM Title Abbreviation: Wirtschaftsdienst
Journal ID: Wirtschaftsdienst
NLM Journal ID: 101086612

Wirtschaftsdienst (Hamburg, Germany : 1949)

ISSN: 0043-6275

ARTICLE INFORMATION
==============================
PMCID: PMC7896181
PMID: 33642651
DOI: 10.1007/s10273-021-2858-9
Article ID: 2858
Article version: 1
Subjects: Ökonomische Trends

Rohstoffpreise unter dem Einfluss von COVID-19

Konjunkturschlaglicht

Economic Headlines: Commodity Prices Under the Influence of COVID-19

Wellenreuther Claudia wellenreuther@hwwi.org

grid.435054.3 0000 0001 2227 8589 HWWI, Oberhafenstraße 1, 20097 Hamburg, Deutschland
Electronic publication date: 2021 Feb 20
Print publication date: 2021
Volume: 101
Issue: 2
First page: 147
Last page: 148
Copyright: © Der/die Autor:in(nen) 2021
License: Open Access: Dieser Artikel wird unter der Creative Commons Namensnennung 4.0 International Lizenz veröffentlicht (http://creativecommons.org/licenses/by/4.0/deed.de).
Open Access wird durch die ZBW–Leibniz-Informationszentrum Wirtschaft gefördert.
License URL: https://creativecommons.org/licenses/by/4.0/

issue-copyright-statement: © The Author(s) 2021

==============================

Die Entwicklungen auf den internationalen Rohstoffmärkten wurden im gesamten Jahr 2020 durch die globale COVID-19-Pandemie dominiert. Die weltweit verhängten Lockdown-Maßnahmen zur Bekämpfung des neuartigen Coronavirus bremsten das globale Wirtschaftswachstum und stürzten die Weltwirtschaft in eine tiefe Rezession. Der durch die COVID-19-Pandemie ausgelöste Schock auf den Rohstoffmärkten beeinflusste die Entwicklungen vieler Rohstoffpreise. Im Gegensatz zu früheren Rezessionen variierten allerdings das Ausmaß und die Richtung der Preisreaktionen je nach Rohstoffgruppe. Während die Preise der Energierohstoffe im Frühjahr im Zuge des ersten Lockdowns stark einbrachen und sich auch zum Ende des Jahres noch deutlich unter dem Vorkrisenniveau bewegten, fielen die Preise für Industriemetalle vergleichsweise moderat und stiegen bis zum Jahresende weit über das Vorkrisenniveau. Da sowohl die Preise für Rohöl als auch für Industriemetalle eng mit der Entwicklung der globalen Weltwirtschaft verbunden sind, stellt deren unterschiedlicher Verlauf eine Besonderheit dar, die beispielsweise in der letzten globalen Rezession ausgelöst durch die Finanzkrise 2008/2009 nicht zu beobachten war.

Abbildung 1 zeigt die Entwicklung der HWWI-Rohstoffindizes[1] für Energie, Industrierohstoffe sowie Nahrungs- und Genussmittel im Jahr 2020. Der Verlauf der Indizes zeigt deutlich, dass insbesondere der erste weltweite Lockdown die Rohstoffmärkte beeinflusste und im Frühjahr zu einem drastischen Einbruch der Energierohstoffpreise führte. Der Index für Energierohstoffe erreichte im April historische Tiefstwerte und lag im Monatsdurchschnitt 27 % unter dem Wert des Vormonats und 53 % unter dem Aprilwert des Vorjahrs. Auch die Preise für Industrierohstoffe sowie für Nahrungs- und Genussmittel fielen bis April, allerdings in einem deutlich geringeren Ausmaß. Im Mai und Juni, im Zuge der Lockerung des ersten globalen Lockdowns, konnten die Preisverluste aller drei Rohstoffindizes größtenteils wieder aufgeholt werden. In der zweiten Jahreshälfte verzeichneten die drei Indizes überwiegend Preisanstiege, trotz eines erneuten globalen Anstiegs der Corona-Infektionszahlen und der Einführung weiterer Lockdown-Maßnahmen. Während die Energiepreise zum Jahresende deutlich unter dem Vorkrisenniveau blieben, stiegen die Preise für Industrierohstoffe ab März so stark an, dass sie zum Jahresende weit über ihrem Vorkrisenniveau lagen.[2]

Diese Unterschiede in den Preisreaktionen der einzelnen Rohstoffsegmente konnten im Zuge der Finanzkrise 2008/2009 nicht beobachtet werden. Hier verzeichneten neben den Energierohstoffen auch die Industrierohstoffe einen starken Preiseinbruch. Darüber hinaus war die Wirkung des Schocks auf den Märkten nach der Finanzkrise persistenter. Die Preise der beiden Rohstoffgruppen wiesen eine längere Erholungsphase auf (vgl. Abbildung 2, World Bank Group, 2020). Der unterschiedliche Verlauf der Energie- und Industriemetallpreise könnte darauf zurückzuführen sein, dass die globale COVID-19-Pandemie und die damit verbundenen Eindämmungsmaßnahmen im Gegensatz zu früheren Rezessionen eine bislang einmalige Kombination von Angebots- und Nachfrageschocks auf den Rohstoffmärkten auslösten, die sich unterschiedlich auf die Rohstoffsegmente auswirkten. Der plötzliche Stillstand der globalen Industrie aufgrund des weltweiten Lockdowns im Frühjahr führte zu einem drastischen

1 Der HWWI-Index für Energierohstoffe umfasst die preislichen Veränderungen von Rohöl, Erdgas und Kohle, der HWWI-Index für Industrierohstoffe die preislichen Veränderungen von industriellen Agrarrohstoffen, Nichteisenmetallen, Eisenerz und Stahlschrott und der HWWI-Index für Nahrungs- und Genussmittel die preislichen Veränderungen von Getreide, Genussmitteln, Ölsaaten und Ölen.

2 Im Dezember notierte der durchschnittliche Kupferpreis fast 30 % höher als der Durchschnitt des entsprechenden Vorjahresmonats, während der Rohölpreis 20 % unter dem Durchschnittspreis vom Dezember 2019 lag.

Abbildung 1
**Entwicklung der HWWI-Rohstoffpreisindizes 2020**
Index auf US-$-Basis, 2015 = 100

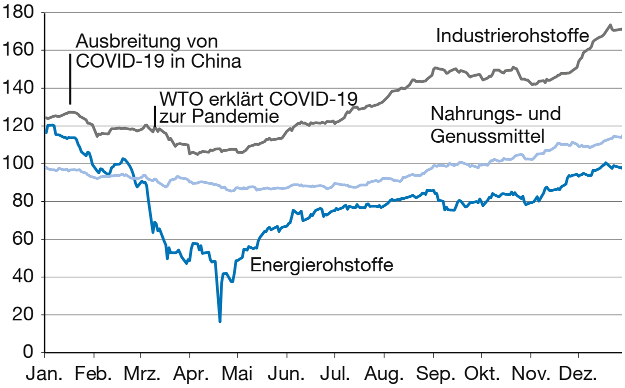

Quelle: Hamburgisches WeltWirtschaftsInstitut (HWWI).

Abbildung 2
**HWWI–Index für Energie- und Industrierohstoffe**
Januar 2020 = 100 bzw. September 2008 = 100

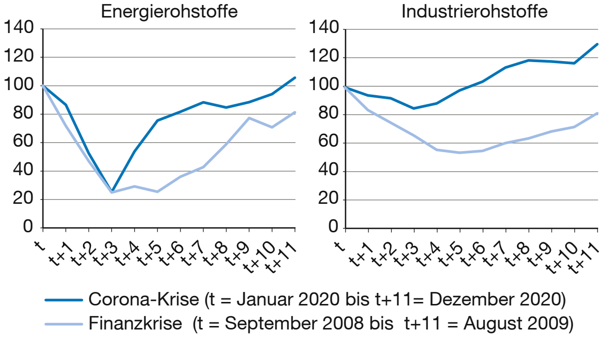

Quellen: Hamburgisches WeltWirtschaftsInstitut (HWWI); World Bank Group (2020).

Nachfrageeinbruch auf den Rohölmärkten. Zusätzlich reduzierten die verhängten Ausgangsbeschränkungen und Grenzsperrungen zunächst in China und anschließend weltweit drastisch den Straßen- und Flugverkehr und somit die Nachfrage nach Treibstoff. Nach Angaben der U.S. Energy Information Administration (2020) sank die weltweite Rohölnachfrage im April auf ein historisches Tief von durchschnittlich 79 Mio. Barrel pro Tag, was einem Nachfragerückgang von 21 Mio. Barrel pro Tag im Vergleich zum April des Vorjahrs entspricht. Der extreme Nachfrageschock in Verbindung mit einer Angebotsausweitung von Russland und Saudi-Arabien, die sich im Frühjahr in einem Preiskrieg befanden, führten im April zu dem drastischen Preiseinbruch auf dem Rohölmarkt. Der ab Mai zu beobachtende steile Aufwärtstrend auf den Rohölmärkten ist einerseits darauf zurückzuführen, dass die Staaten der OPEC+ ihre Rohölförderung in historischem Ausmaß drosselten, um den Ölpreis zu stabilisieren. Andererseits kurbelte die sukzessive Lockerung der globalen Lockdown-Maßnahmen die Rohölnachfrage wieder an.

Im Gegensatz zu den Rohölmärkten reduzierten die weltweit verhängten Lockdown-Maßnahmen im Frühjahr nicht nur die Nachfrage nach, sondern auch das Angebot an Industriemetallen. Neben Nachfragerückgängen durch die gebremste Industrieproduktion, insbesondere in China, führten die strengen Lockdown-Maßnahmen in den Förderländern zu temporären Minenschließungen und damit zu einer Verknappung des Angebots. Diese Verknappung milderte den Preisverfall auf den Märkten für Industriemetalle im Frühjahr. Der starke Preisanstieg auf den Industriemetallmärkten, der sich seit Mai trotz der schwachen Weltkonjunktur kontinuierlich fortsetzte, verdeutlichte die enorme Bedeutung Chinas, dem weltweit größten Verbraucher von Industriemetallen. Zusätzlich zeigt der Anstieg, dass es der chinesischen Wirtschaft im Vergleich zu anderen großen Volkswirtschaften schneller gelungen ist, sich von der Rezession zu erholen. Während viele Länder insbesondere zum Jahresende mit steigenden Corona-Infektionen kämpften, scheint es China bereits im Frühjahr gelungen zu sein, das Virus erfolgreich einzudämmen. Unterstützt durch staatliche Konjunkturprogramme führte die schnelle Erholung der chinesischen Wirtschaft zu einem massiven Anstieg der Nachfrage auf den Märkten für Industriemetalle.

Die Preise für Nahrungs- und Genussmittel wurden von der Corona-Krise nur geringfügig beeinflusst. Die Lockdown-Maßnahmen lösten auf diesen Märkten zwar ebenfalls eine Kombination aus Angebots- und Nachfrageschocks aus, die jedoch lediglich zu moderaten Preisbewegungen führten. Beispielsweise ging der Preisverfall für Rohöl im Frühjahr mit einem Einbruch der Biokraftstoffproduktion einher, was wiederum zu einem Nachfragerückgang bei Mais, Zucker und Pflanzenölen führte. Zum Jahresende verzeichneten die Pflanzenölmärkte hingegen steigende Preise aufgrund eines verknappten Angebots. Arbeitskräftemangel in den Anbauländern aufgrund strikter Lockdown-Maßnahmen führte im Frühjahr zu einem Rückgang des Anbaus an Ölpalmen. Auf den Märkten für Grundnahrungsmittel wie Gerste, Weizen und Reis kam es im Frühjahr zu einem Anstieg der Nachfrage bei gleichzeitiger Angebotsverknappung. Um befürchteten Nahrungsmittelengpässen vorzubeugen, erhöhten viele Länder die Nachfrage nach Grundnahrungsmitteln. Zudem schränkten die weltweit verhängten Ausgangssperren die Verfügbarkeit von Arbeitskräften ein, was zu Schwierigkeiten bei der Ernte dieser Rohstoffe führte.

Im Gegensatz zu früheren Rezessionen wirkte sich der durch die Corona-Krise ausgelöste Schock in unterschiedlichem Maße auf die Rohstoffmärkte aus. Es bleibt abzuwarten, ob der Anstieg des Verbrauchs von Industriemetallen bei gleichzeitigem Rückgang des Rohölverbrauchs eine strukturelle Veränderung widerspiegelt, die sich auch langfristig auf den Rohstoffmärkten bemerkbar macht. Die zunehmende Digitalisierung und der durch die Corona-Krise unterstützte Trend zur Remote-Arbeit könnten beispielsweise auch langfristig die Konsumentscheidungen beeinflussen und über die Krise hinaus zu einem Rückgang an Flugreisen führen. Zudem könnten staatliche Konjunkturprogramme in Form von klimafreundlichen Investitionen den 2020 beobachteten Trend auf den Rohstoffmärkten unterstützen.

Claudia Wellenreuther
wellenreuther@hwwi.org


Literatur

U.S. Energy Information Administration (2020), Short-Term Energy Outlook, eia.gov/outlooks/steo/archives/jul20.pdf (4. Januar 2021).
World Bank Group (2020), Commodity Markets Outlook, Oktober, open-knowledge.worldbank.org/bitstream/handle/10986/34621/CMO-October-2020.pdf (4. Januar 2021).
